# Diagnosis and Treatment Approach for Giant Muscle Hematoma of the Thigh Due to a Post-traumatic Arteriovenous Fistula: A Case Report

**DOI:** 10.7759/cureus.102420

**Published:** 2026-01-27

**Authors:** Takahiro Tanabu, Shusa Ohshika, Tatsuro Saruga, Yuki Fujita, Shinya Kakehata, Shingo Kakeda, Yasuyuki Ishibashi

**Affiliations:** 1 Orthopedic Surgery, Hirosaki University Graduate School of Medicine, Hirosaki, JPN; 2 Radiology, Hirosaki University Graduate School of Medicine, Hirosaki, JPN

**Keywords:** embolization, hematoma, post-traumatic arteriovenous fistula, signal void, surgical resection

## Abstract

Intramuscular hematomas have varied etiologies, and accurate diagnosis requires a detailed medical history in combination with laboratory and imaging evaluations. We report a case of a giant thigh hematoma that enlarged over a chronic course and was ultimately attributed to a post-traumatic arteriovenous fistula (AVF).

An 18-year-old man was diagnosed with an intramuscular hematoma after blunt trauma to the thigh, which gradually enlarged. He later presented with sudden worsening of thigh swelling and pain and was transported to the emergency department. Contrast-enhanced CT showed extravasation in the proximal thigh together with dilation of the internal iliac and inferior gluteal arteries, raising suspicion of a vascular malformation. Angiography performed the same day confirmed active bleeding from an AVF at the level of the inferior gluteal artery. Because surgical hemostasis was deemed difficult, transcatheter arterial embolization (TAE) was performed, and achieved hemostasis. Three months later, with no recurrence of abnormal vessels, the giant hematoma was excised. The patient has remained free of recurrence for five years. When encountering a large intramuscular hematoma, clinicians should consider a high-flow vascular malformation. Identification of signal voids on MRI and extravasation on contrast-enhanced CT is critical for early diagnosis. For active bleeding due to high-flow lesions, arterial embolization is an effective therapeutic option.

## Introduction

Intramuscular hematoma is occasionally encountered in daily practice. It may follow high-energy trauma such as sports injuries or traffic accidents, or arise after minor trauma in patients with bleeding diathesis, including hemophilia, liver cirrhosis, or anticoagulant use [1]. Neoplastic lesions (e.g., soft-tissue metastases, sarcomas) and vascular lesions (e.g., vascular malformations, aneurysms) can also lead to hematoma formation [2]. Accordingly, diagnosis relies on detailed history taking (mechanism of injury, comorbidities), assessment for coagulation abnormalities, and careful interpretation of CT and MRI [3].

In the acute phase, conservative management with rest, ice, compression, and elevation (RICE) is standard. When a hematoma persists and organizes, surgical excision may be indicated in rare cases [4]. Although surgical management of chronic expanding hematoma has been reported frequently, formal clinical guidelines remain unclear, and treatment decisions are typically individualized.

Here, we describe a case initially considered a chronic expanding hematoma of the thigh that chronically enlarged but proved to be caused by a post-traumatic arteriovenous fistula (AVF). We discuss diagnostic points and therapeutic strategies for large intramuscular hematomas.

## Case presentation

An 18-year-old man struck the posterior aspect of his left thigh while riding a bicycle and visited a local hospital. MRI showed a poorly demarcated, heterogeneous mass measuring up to 20 cm in diameter in the posterior thigh, with mixed low- and high-signal intensity on both T1- and T2-weighted images. He was diagnosed with an intramuscular hematoma and managed conservatively. Eight months later, swelling of the left posterior thigh recurred without provocation. MRI demonstrated interval enlargement compared with the initial study. Laboratory tests showed that Factor VIII and Factor IX activities were normal at 100.7% and 105.5%, respectively, with negative inhibitor assays (<0.5 BU/mL), and prothrombin time-international normalized ratio (PT-INR) (1.07) and activated partial thromboplastin time (APTT) (30.9 s) were also within normal limits. Needle biopsy confirmed hematoma. Given the clinical course and minimal symptoms, the lesion was observed as a chronic expanding hematoma [5].

**Figure 1:**
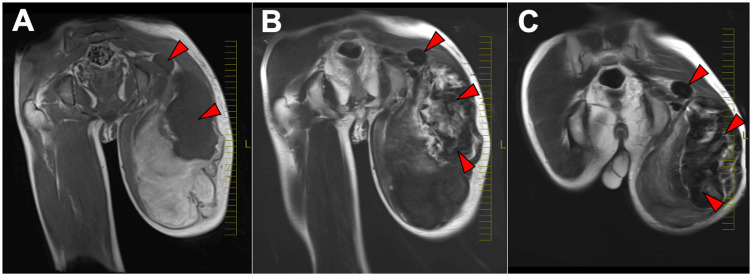
Coronal MRI obtained at presentation to the emergency room: (A) T1-weighted, (B) T2-weighted, and (C) contrast-enhanced T1-weighted images. A large heterogeneous mass with mixed high- and low-signal intensities extends from the left gluteal region to the posterior thigh. Signal-void areas (panels A-C, red arrows), consistent with a high-flow vascular component, are observed in the gluteal region.

Eighteen months after the initial injury, swelling and pain recurred, so he was transported to the emergency department. MRI revealed a giant (30 cm) poorly defined mass extending from the gluteal region to the posterior thigh, again with mixed signal intensities and an area in the gluteal region suggestive of flow voids (Figures 1A-1C).

**Figure 2:**
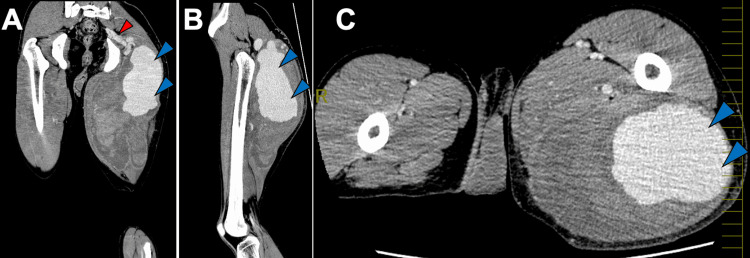
Contrast-enhanced CT obtained on arrival at the emergency room: (A) coronal, (B) sagittal, and (C) axial views. Extensive contrast extravasation (panels A-C, blue arrows) within the posterior proximal thigh hematoma, indicating acute bleeding, is observed. Marked dilation of the internal iliac artery and inferior gluteal artery (panel A, red arrow), appearing continuous with the lesion, is also noted.

On arrival, he was tachycardic (heart rate: 108 bpm) and hypotensive (systolic blood pressure: 90 mmHg), and blood tests showed anemia (hemoglobin: 8.5 g/dL). Contrast-enhanced CT demonstrated extensive contrast extravasation within the proximal posterior thigh hematoma compatible with acute bleeding/pseudoaneurysm (Figures 2A-2C).

**Figure 3:**
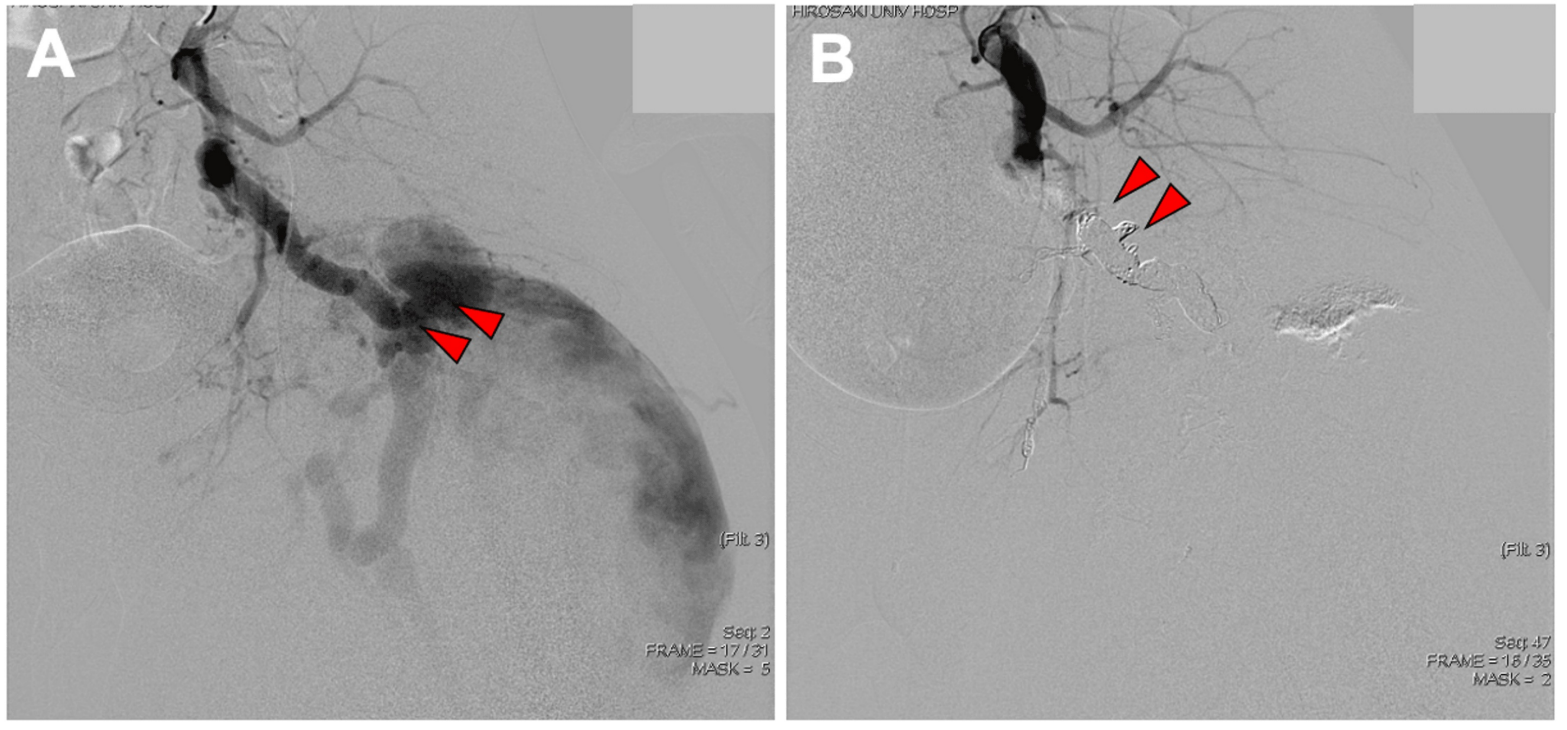
DSA images obtained (A) before and (B) after transcatheter arterial embolization. Initial angiography of the internal iliac artery demonstrated active bleeding from an arteriovenous fistula (panel A, red arrows) at the level of the inferior gluteal artery. Combined coil and NBCA embolization successfully occluded the inflow (panel B, red arrows) to the pseudoaneurysm. DSA: digital subtraction angiography; NBCA: n-butyl-2-cyanoacrylate

**Figure 4:**
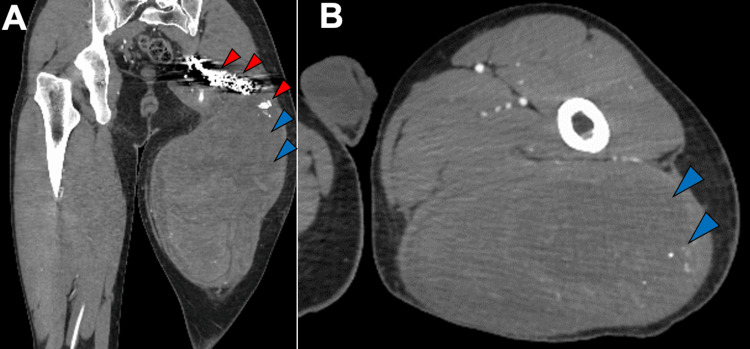
Contrast-enhanced CT obtained three months after the third transcatheter arterial embolization: (A) coronal and (B) axial views. No abnormal vessels or contrast extravasation are detected (panel A and B, blue arrows) within or around the hematoma. Multiple embolization coils (panel A, red arrows) from prior procedures are visible.

Marked dilation of the inferior gluteal and internal iliac arteries suggested a vascular malformation. Angiography on the same day confirmed active bleeding from an AVF at the inferior gluteal artery level (Figure 3A). TAE using coils and n-butyl-2-cyanoacrylate (NBCA) was performed three times-on the day of admission, the following day, and seven days later-resulting in complete occlusion of flow into the pseudoaneurysm (Figure 3B). Three months after the final embolization, contrast-enhanced CT showed no abnormal vessels or extravasation, and the hematoma size was unchanged (Figures 4A, 4B).

Surgical excision was therefore undertaken. Under general anesthesia in the prone position, a posterior approach to the thigh was used, and the encapsulated hematoma was removed en bloc while preserving surrounding nerves and muscles. Operative time was 3 h 35 min; intraoperative blood loss was 360 mL. The specimen weighed 2400 g and was histologically confirmed as an organized hematoma. Contrast-enhanced MRI at one year showed no recurrence or abnormal vasculature. The patient remains recurrence-free five years after surgery.

## Discussion

We experienced a rare case of a giant thigh hematoma that chronically enlarged due to an AVF. When encountering a patient with a hematoma, it is crucial to identify the bleeding source by checking for signal voids on MRI and contrast extravasation on contrast-enhanced CT. When a hematoma is determined to be caused by a high-flow vascular malformation, such as an arteriovenous malformation (AVM) or AVF, initial management should focus on hemostasis via TAE, followed by assessment of the vascular lesion and consideration of surgical removal of the residual hematoma.

**Figure 5:**
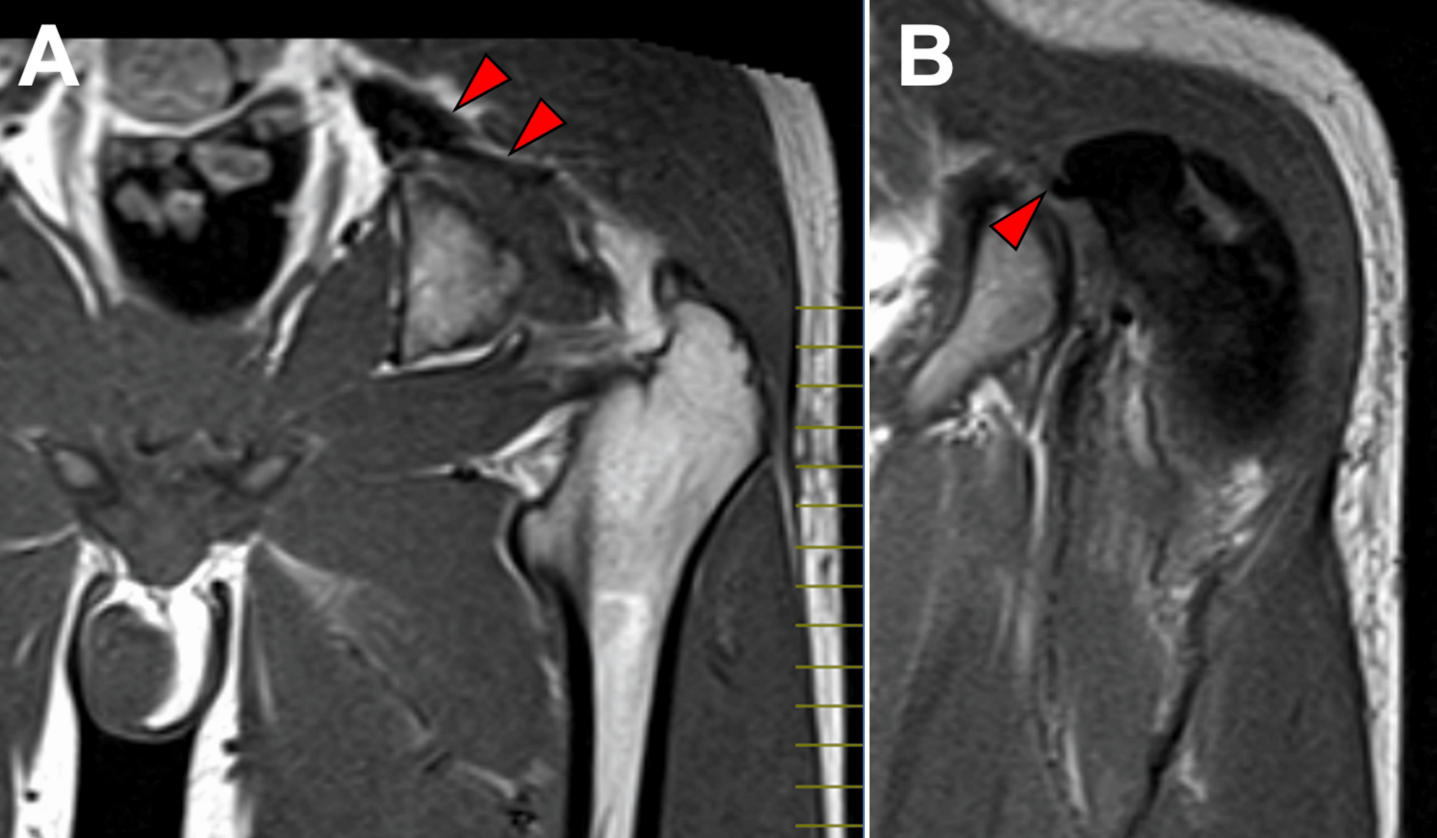
Initial MRI at the referring hospital: coronal T1-weighted images. Dilation of the left internal iliac artery extending to the inferior gluteal artery (panel A, red arrows) and a contiguous intramuscular signal-void area (panel B, red arrow) within the left gluteus maximus is observed.

When interpreting MRI in cases diagnosed as a hematoma, careful attention should be paid not to overlook signal voids, which may indicate the bleeding source. In our case, retrospective review of the initial MRI obtained at the referring hospital revealed dilation of the internal iliac and inferior gluteal arteries and a contiguous signal void within the gluteus maximus (Figures 5A, 5B).

However, signal voids can appear as low-intensity areas on MRI and may be misinterpreted as organized hematoma. In this case, the vascular anomaly was initially missed, and the lesion was subsequently managed as a chronic, expanding hematoma. When a signal void is suspected on MRI, contrast-enhanced CT can help confirm extravasation and identify the bleeding source [5], thereby distinguishing it from an organized hematoma. In relatively superficial lesions, color Doppler ultrasonography may also be useful for detecting arterial flow [6], which can suggest the presence of a high-flow vascular malformation [7,8].

When a hematoma is caused by a high-flow vascular malformation, TAE should be the first-line treatment to control active bleeding. Diagnostic flowcharts and treatment algorithms for vascular malformations have been described in Japanese clinical guidelines, but given the clinical heterogeneity (age, location, lesion type, and size), treatment decisions should be individualized [9]. Management options include conservative therapy, arterial embolization, sclerotherapy, surgical resection, or a combination of these approaches [10].

In our case, the patient presented with acute bleeding and hypotension that required urgent intervention. Because of the markedly dilated internal iliac artery and the deep, proximal location of the bleeding source, surgical hemostasis was considered hazardous and technically difficult; therefore, TAE was selected. If the bleeding source had been located more distally and superficially in the limb, surgical control might have been feasible using a tourniquet. Complete hemostasis was achieved after three embolization sessions, resulting in improvement of the internal iliac artery dilation.

There is currently no established guideline or evidence-based consensus regarding the optimal timing of follow-up evaluation after endovascular treatment for arteriovenous fistulas. Previous reports have described variable follow-up intervals depending on lesion characteristics and clinical course [11], with several case reports performing mid-term reassessment at approximately three months after the initial intervention [12,13]. Such follow-up enables confirmation of complete hemostasis and early detection of residual or recurrent shunting that may require additional treatment. As no spontaneous reduction in hematoma size was expected, surgical excision of the residual organized hematoma was subsequently performed, leading to complete recovery.

## Conclusions

We reported a case of a giant thigh hematoma that chronically enlarged due to an AVF. Because standardized guidelines for managing intramuscular hematomas are lacking, clinicians should obtain a thorough history (including mechanism of injury, comorbidities, and anticoagulant use), perform laboratory testing (platelet count, PT, APTT), and obtain multimodal imaging (contrast-enhanced CT, MRI, ultrasonography) to differentiate neoplastic from vascular causes. In cases of hematomas that enlarge over time, clinicians should consider not only chronic expanding hematoma and malignant tumors but also high-flow vascular malformations. Identifying signal voids on MRI and extravasation on CT is essential for early and accurate diagnosis. When active bleeding is caused by a high-flow vascular malformation, initial treatment with arterial embolization followed by surgical excision of the residual hematoma is an effective therapeutic strategy.
